# Supplementary material for: Causal Relationship between Aging and Anorexia Nervosa: A White-Matter-Microstructure-Mediated Mendelian Randomization Analysis
Source: Biomedicines. 2024 Aug 16;12(8):1874. doi: 10.3390/biomedicines12081874 (PMC11351342; doi:10.3390/biomedicines12081874)
Supplement: Supplementary file 1 [file biomedicines-12-01874-s001.zip › Supplementary Figure/Figure S4/Singe SNP plot of MR analysis for TL to ubm-b-1981.pdf]

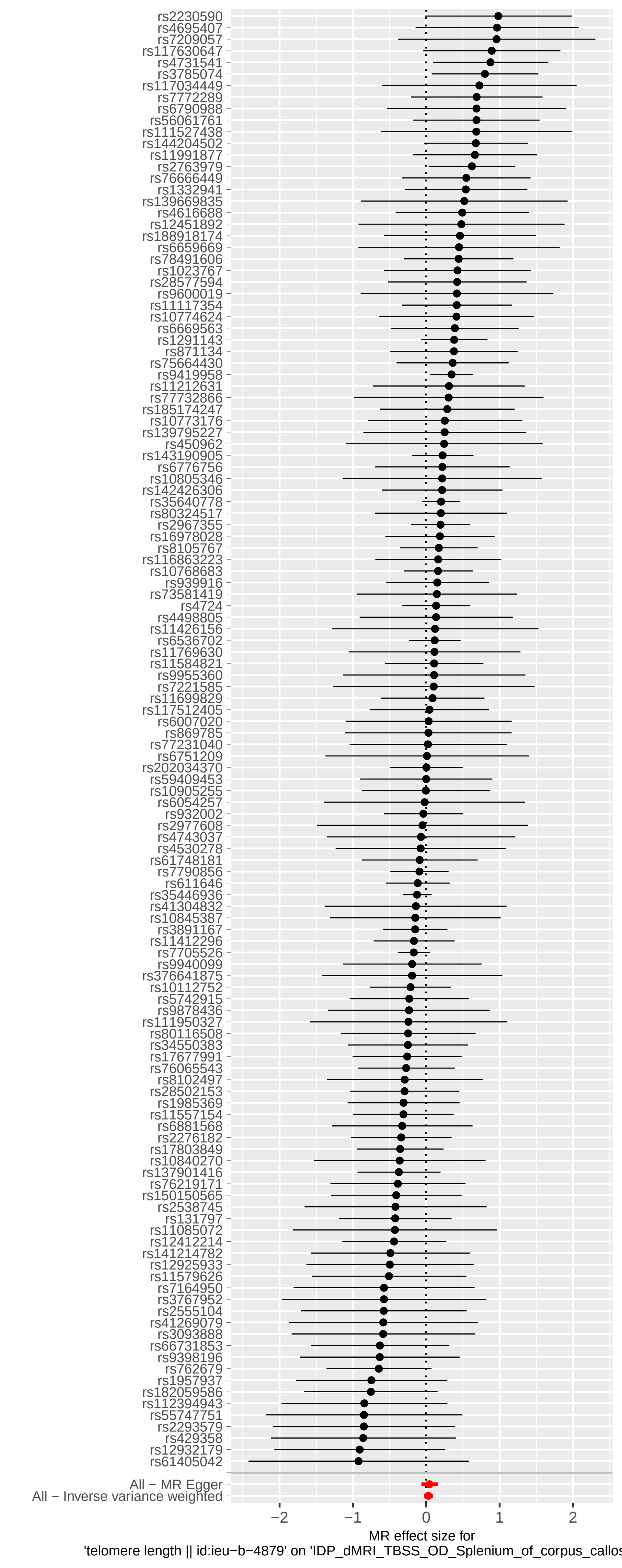
rs2230590
rs4695407
rs7209057
rs117630647
rs4731541
rs3785074
rs117034449
rs7772289
rs6790988
rs56061761
rs111527438
rs144204502
rs11991877
rs2763979
rs76666449
rs1332941
rs139669835
rs4616688
rs12451892
rs188918174
rs6659669
rs78491606
rs1023767
rs28577594
rs9600019
rs11117354
rs10774624
rs6669563
rs1291143
rs871134
rs75664430
rs9419958
rs11212631
rs77732866
rs185174247
rs10773176
rs139795227
rs450962
rs143190905
rs6776756
rs10805346
rs142426306
rs35640778
rs80324517
rs2967355
rs16978028
rs8105767
rs116863223
rs10768683
rs939916
rs73581419
rs4724
rs4498805
rs11426156
rs6536702
rs11769630
rs11584821
rs9955360
rs7221585
rs11699829
rs117512405
rs6007020
rs869785
rs77231040
rs6751209
rs202034370
rs59409453
rs10905255
rs6054257
rs932002
rs2977608
rs4743037
rs4530278
rs61748181
rs7790856
rs611646
rs35446936
rs41304832
rs10845387
rs3891167
rs11412296
rs7705526
rs9940099
rs376641875
rs10112752
rs5742915
rs9878436
rs111950327
rs80116508
rs34550383
rs17677991
rs76065543
rs8102497
rs28502153
rs1985369
rs11557154
rs6881568
rs2276182
rs17803849
rs10840270
rs137901416
rs76219171
rs150150565
rs2538745
rs131797
rs11085072
rs12412214
rs141214782
rs12925933
rs11579626
rs7164950
rs3767952
rs2555104
rs41269079
rs3093888
rs66731853
rs9398196
rs762679
rs1957937
rs182059586
rs112394943
rs55747751
rs2293579
rs429358
rs12932179
rs61405042
All − MR Egger
All − Inverse variance weighted
−2
−1
0
1
2
MR effect size for
'telomere length || id:ieu−b−4879' on 'IDP_dMRI_TBSS_OD_Splenium_of_corpus_callos
